# The Deep Roots of the Rings of Life

**DOI:** 10.1093/gbe/evt194

**Published:** 2013-11-26

**Authors:** James A. Lake, Janet S. Sinsheimer

**Affiliations:** ^1^Department of MCD Biology, University of California, Los Angeles; ^2^Department of Human Genetics, University of California, Los Angeles; ^3^Department of Biostatistics, University of California, Los Angeles; ^4^Department of Biomathematics, University of California, Los Angeles

**Keywords:** photosynthesis, ether lipids, sporulation, indels, genomes, hypergeometric distributions

## Abstract

Reconstructing early evolutionary events like the origins of informational and operational genes, membranes, and photophosphorylation is difficult because early evolutionary events can be masked by subsequent gene flows. Furthermore, as evolution progresses through both Darwinian survival of the fittest (tree-like evolution) and symbiotic/endosymbiotic cooperation (ring-like evolution), trees alone are not adequate to represent Earth’s evolutionary history. Here, we reconstruct and root the New Rings of Life and use it as a framework for interpreting early events in the evolution of life. Unlike the three-domain hypothesis, the rings do not fit all life into one of three immutable categories, but rather accommodate new gene flows as novel organisms are discovered. A draft of the Rooted Rings of Life is reconstructed by analyzing the phylogenetic distributions of indels (insertions/deletions) and genes coding for fundamental molecular processes. Their phylogenetic distributions are inconsistent with all trees. Hypergeometric distribution analyses of them strongly localize the root of the rings to a segment of the deepest ring (*P* < 10^−21^ and *P* < 10^−194^), and whole-genome analyses independently confirm the topology of the rooted rings (*P* < 7.1 × 10^−6^). The rings identify several large gene flows, including a flow of a thousand genes into the *Halobacteria* and the *Eubacteria*, the related photocyte flow, the flow of genes into the last common ancestor of the eocytes and the eukaryotes, and the informational and operational gene flows into the eukaryotes. The rooted rings also chronologically order steps in the evolution of extant taxa, that is, phototrophy evolved from *Halobacteria* (photophosphorylation) → *Heliobacteria* (photosynthesis) → *Cyanobacteria* (oxygenic photosynthesis).

## Introduction

**Fig. 1.—:**
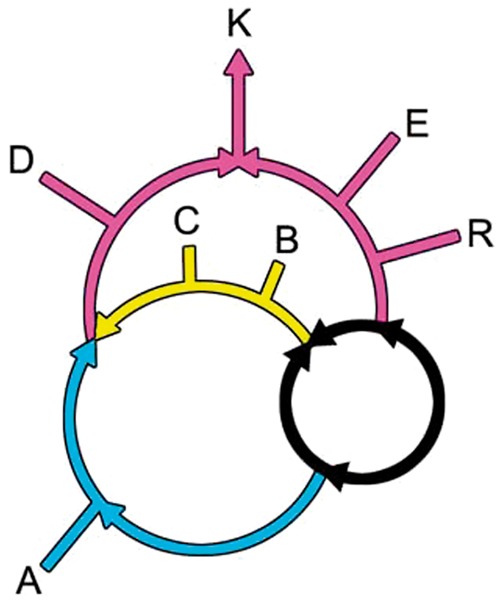
The rooted rings of life consist of an outer, a middle, and an inner ring. The outer ring, shown in pink, resulted in the origin of the eukaryotes (K) through gene flows from the DM prokaryotes (D) on the left and from the eocytes (E) on the right (Rivera and Lake 2004). The DM prokaryotes (D) originated in the middle ring when gene flows from the *Actinobacteria*, shown in blue, and the *Bacilli* and the *Clostridia*, shown in yellow, endosymbioticaly converged (Skophammer et al. 2007; Lake 2009). The previously unknown inner ring, shown in black, contains the root of the rings of life.

Our current knowledge of the topology of the rings of life is summarized in figure 1. An outer eukaryotic ring (Rivera and Lake 2004), shown in pink, relates the origin of eukaryotes (K) to two converging large gene flows from the double-membrane (DM) (Gram-negative) prokaryotes (D) and from the eocytes (E) (Lake et al. 1984; Rivera and Lake 2004) and the *Euryarchaeota* (R). Within a second ring, gene flows from the *Actinobacteria* (A), shown in blue, and from the *Firmicutes* (the *Clostridia*, C, and the *Bacilli*, B), shown in yellow, converge to form the DM prokaryotes. Within these two rings, a previously unexplored central system of inner rings schematically represented by a black ring connects the root of life to the two outer rings.

In the outer ring, the eukaryotes arise when a gene flow from euryarchaea (R) into the eocyte prokaryotes (E) converges with the flow from the DM prokaryotes. This is thought to be due to a symbiotic/endosymbiotic merger, based on whole-genome presence/absence studies (Rivera and Lake 2004). Recently, this large flow of informational genes from the eocytes into eukaryotes has received strong statistical support in numerous, sophisticated multi-gene tree reconstructions (Archibald 2008; Cox et al. 2008; Poole and Neumann 2011; Williams et al. 2012).

Within the second ring, separate gene flows from the *Actinobacteria* and the *Firmicutes* fuse to produce the DM or Gram-negative prokaryotes (Lake 2009). This genome fusion is consistent with, but does not prove that, an endosymbiotic origin produced the inner and outer membranes of the DM prokaryotes. However, the flows in this analysis provide some additional information about this process.

A previously unknown prokaryotic inner ring, shown in black, connects the roots of life to the gene flows leading to the DM ring and to the extended eocytes. This inner ring relates the five prokaryotic super-taxa that encompass known prokaryotic life (Boone and Castenholz 2001; Skophammer et al. 2007). These five are the *Actinobacteria*, A, the DM prokaryotes, D; the *Firmicutes*, F, the *Euryarchaeota,* R, and the *Eocyta* (E) (Lake 1984), the sister taxon of the eukaryotes in the informational gene flow (Rivera et al. 1998).

Together, these groups encompass prokaryotic life on Earth. The DM prokaryotes, D, contain all known photosynthetic prokaryotes, except for the photosynthetic *Clostridia* (the *Heliobacteria*), and numerous nonphotosynthetic species as well. Within the DM prokaryotes, evolution is highly non-tree-like (Garrity and Holt 2001), suggesting that rings are common within this group. The Gram-positive *Actinobacteria* (on the blue lineage) are characterized by having high GC DNA compositions and contain both free-living and pathogenic species, including those responsible for leprosy and tuberculosis. The *Firmicutes*, shown on the yellow portion of this inner ring, are represented by the *Clostridia* and *Bacilli*. *Firmicutes* are unique in containing endospores, cells within cells, that can remain dormant for extended periods (Errington 2003; Higgins and Dworkin 2012). The *Euryarchaeota* consist of halophiles, methanogens, and related organisms, and the *Eocyta* are the prokaryotic sister taxon to the eukaryotes (Williams et al. 2012).

Using genome analyses, we reconstruct and root the central rooted ring based on indels and whole genomes and relate the central ring to the outer rings. These analyses strongly support the rooted ring topology presented here.

## Materials and Methods

Reconstructing the evolution of the central black ring is greatly facilitated by the analyses of duplicated essential genes. The gene sets analyzed here have the remarkable property that they are phylogenetically distributed so that no tree can explain them. Although they do not support any tree, they do support a unique rooted ring. Some of these duplicated genes have been extensively documented in prior, tree-based indel analyses (Skophammer et al. 2006; Servin et al. 2007; Skophammer et al. 2007; Lake et al. 2008a, 2008b; Lake et al. 2009), but it has not been previously recognized that they contradicted all possible rooted trees. Ultimately, the conflicts present within these genes led us to reconstruct the unique central rooted ring reported here.

Indel analyses utilize the phylogenetic patterns of insertions and deletions within duplicated genes coding for fundamental processes (Servin et al. 2007; Lake et al. 2008a, 2008b). As used here to root rings, they can identify unique roots accurately and reliably (*P* < 10^−^^20^). In contrast to gene presence/absence methods, these methods require indel containing duplicated genes which are relatively uncommon. Here, both indel and gene presence/absence methods are used for rooting and determining the topology of the inner black ring.

The process of rooting trees and rings with indels is illustrated using the duplicated, orthologous gene pairs ParC/GyrA and HisF/GGGPS. ParC is a topoisomerase (Servin et al. 2007), proteins that relieve the topological strains encountered by DNA molecules during replication, transcription, and recombination. The phylogenetic distributions of ParC and of its orthologous partner GyrA, which also helps untangle DNA, are shown in table 1.

**Table 1:** ParC Genes are Absent from Euryarchaeota and Eocyta

**ParC**	
D-Proteobacteria	NGAGGIAVGMATNIPPHNLGEVIDACLLLIDQPDVT----TDQLLDLVPGPDFPT
D-Proteobacteria	NGTTGIAVGMATDIPPHNLREVAQAAIALIDQPKTT----LDQLLDIVQGPDYPT
D-Cyanobacteria	NGCSGIAVGMATNVPPHNLGEVVDGLIALIDNPDLP----DEKLFQLIPGPDFPT
A-Actinobacteria	NGASGIAVGMATNMAPHNLVEVVGAARHLLDNPDAT----LDDLMAYIPGPDLPS
F-Firmicutes	NGANGIAVGMTTNIPPHNLSEVISGLHMLMRNPDAT----TKDLMKEIPGPDFPT
R-Euryarchaeota	**--------------- **Genes Absent ----------------
E-Eocyta	** --------------- **Genes Absent ----------------
**GyrA**	
D-Proteobacteria	NGSGGIAVGMATNIPPHNLGEVIDGCVALIDNPAIE----LSELMELIPGPDFPT
D-Proteobacteria	NGSSGIAVGMATNIPPHNLTEVINGCLAYIDDEDIS----IEGLMEHIPGPDFPT
D-Cyanobacteria	NGSSGIAVGMATNIPPHNLGELIDALVAVIHNPEIT----DLELMQYVHGPDFPT
A-Actinobacteria	NGSSGIAVGMATNIPTHNLREVNEAVQWSLAHPNASHEELLEACMERIKGPDFPG
F-Firmicutes	NGSTGISSGYATEIPPHNLGEVIDATIYLLKHPNAS----LEDLMNYVKGPDFPT
R-Archaebacteria	NGSSGIAVGMSTNIPPHNLGELVDATVHLLGNPDCT----VEDLMEHIKGPDFPT

ParC is present in *Actinobacteria*, DM prokaryotes (A, D), and *Firmicutes*, represented by the *Bacilli* and the *Clostridia* (B, C); but it is absent from the archaebacteria, represented by the *Eocyta* (E) and the *Halobacteria* (H). In contrast, its paralogous partner, GyrA, is present in all taxa (A, D, B, C, E, H).

A second gene, Gerenylgerenylglyceryl Phosphate Synthase or GGGPS, is the terminal member of the ether lipid biosynthesis pathway, and HepBP is the product of this pathway. HepBP has long been known to be present in archaea and has recently been detected in the *Firmicutes*. It is described as “… the first archaea-type G1P-based ether lipid being identified within the phylogenetic domain of the *Bacteria*, … ” (Guldan et al. 2011). Thus, both GGGPS and its gene product HepBP are present in archaea (Boucher et al. 2004) and the *Firmicutes* (Guldan et al. 2011).

The phylogenetic distributions of GGGPS and its orthologous partner HisF, an essential gene within the histidine biosynthesis pathway, are shown in table 2. GGGPS and HisF are both members of the beta/alpha barrel family (supplementary section S2, Supplementary Material online). GGGPS genes are missing from the *Actinobacteria* and the DM prokaryotes, whereas HisF genes are present in all prokaryotes.

**Table 2:** GGGPS Genes are Absent from DM Prokaryotes and Actinobacteria

**HisF**	
D-Proteobacteria	KNGFDLGVTRAISDALGIPVIASGGVGNLQH
D-Proteobacteria	KSGFDLELTRAVSDAVPVPVIASGGVGNLQH
D-Cyanobacteria	QAGYDLELTRAVAQAVPVPVIASGGAGCLDH
D-Deino-Thermus	RAGFDLEATRAVAREVDLPVIASGGAGKVQD
D-Other DM Proks	KDGYDIELNRAISEAVNIPVIASGGAGKKEH
A-Actinobacteria	KAGFDLALLRAVRAAVTVPVIASGGAGAVEH
F-Bacilli	KNGYDLRLTEEISKSVSVPVIASGGCGHADH
F-Clostridia	KDGYDIELTRTVSENVKIPVIASGGAGKMEH
R-Halobacteria	KDGYDIPLMKAVCDTVSTPVIASSGCGSPED
R-Thermoplasmata	KKGFDTDLIRKITGSVNIPVIASGGAGSPED
R-Thermoprotei	RLGYDLELTRKIVDSVNIPVIASGGAGKMEH
**GGGPS**	
D-Proteobacteria	-------------------------------
D-Proteobacteria	-------------------------------
D-Cyanobacteria	-------------------------------
D-Deino-Thermus	--------- Genes absent ---------
D-Other DM Proks	-------------------------------
A-Actinobacteria	-------------------------------
B-Bacilli	MLGDIEAVKKTKAVLETSTLFYGGGIKDAET
C-Clostridia	RFGDPAWVGAAAGAMRGARLFYGGGIGTAEQ
B-Bacilli	IYGDVSKVQAVSEHLTETQLFYGGGISSEQQ
R-Halobacteria	MFGDTEKVQAAHDALDDATLFYGGGIRDYDA
R-Archaeoglobus	IYGNPELVAEVKKVLDKARLFYGGGIDSREK
R-Methanobacter	PEHVPEEMIALVKRCTDQILIVGGGIRSGED
E-Methanocaldoc	SYPVNNETIALSKKLSGINIIVGGGIRKPEI
E-Pyrococcus	PEPVPEEMVRVVKSVIDVPLIVGGGIKSGEQ

**Fig. 2.—:**
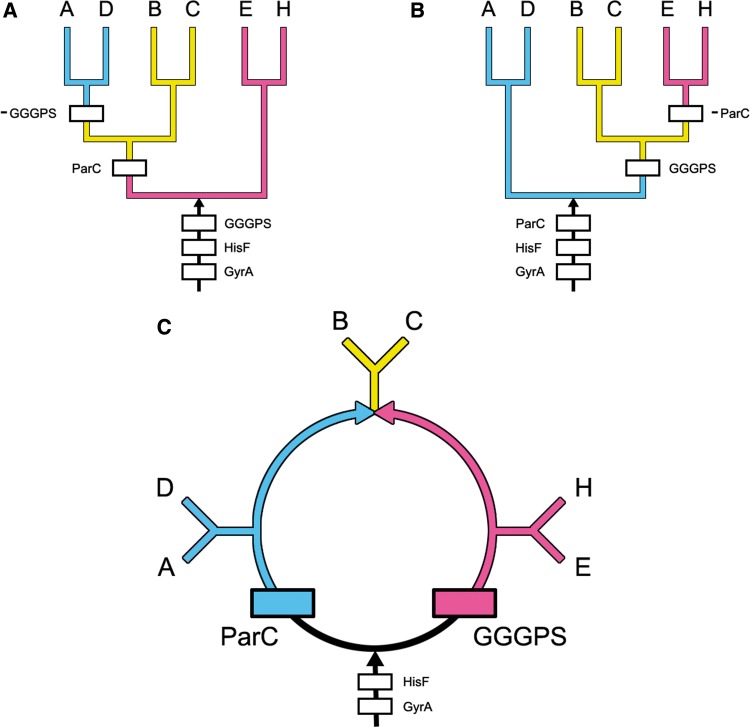
The phylogenetic distribution of the ParC, GGGPS, HisF, and GyrA gene flows are shown in the most parsimonious trees and rings that represents the evolution of the *Actinobacteria* (A), the DM prokaryotes (D), the *Halobacteria* (H), the *Eocyta* (E), and the *Firmicutes*, i.e., the *Bacilli* (B) and the *Clostridia* (C). (*A* and *B*) Trees that most parsimoniously explain the distributions of these genes. (*C*) Ring that most parsimoniously explains their evolution. As discussed in the text, both trees (shown in *A* and *B*) require four gene gain sites and one gene loss site. In addition, two gene gain sites, HisF and GyrA, are present within the roots leading to both of these trees. The two other gene gain sites, marked by boxes labeled ParC and GGGPS, are present either within the trees or within the root. In addition, one gene loss site is present within each tree. These gene loss sites are marked by -GGGPS and by -ParC in (*A*) and (*B*), respectively. The rooted ring shown in (*C*) defines the evolution of these six major prokaryotic groups more parsimoniously and much more likely than either tree. Here, the root is represented by the black arrow at the bottom. The origin and flow of ParC genes into A and D is shown in blue; and the origin and flow of GGGPS genes into H and E are shown in pink. Where both genes converge and flow into the *Firmicutes*, B and C, they are shown in yellow.

It is well known that trees can be rooted by analyzing duplicated genes and less well known that rings can also be rooted using the same reasoning. Here, we apply this reasoning to rooting rings as well. In figure 2*A*, the origin of the ParC gene is most parsimoniously explained by a gene duplication from GyrA (marked by the box labeled ParC). Beyond this point, the orthologous gene flows representing ParC and GyrA and HisF are free to flow into A, D, B, and C. As the GGGPS gene is absent from A and D, a loss of GGGPS genes is required to prevent them from flowing into A and D. This gene loss (or stop) site is marked -GGGPS in figure 2*A*.

Similarly in figure 2*B*, a ParC gene loss site, marked -ParC, is required to prevent ParC genes from flowing into E and H. Hence, both rooted trees in figure 2*A* and *B* require four gene gain (or start) sites (GGGPS, HisF, ParC, and GyrA) and one highly unlikely, -ParC or -GGGPS, gene loss site. The seven remaining rooted trees (not shown) require four gene gain sites and one or more gene loss sites.

In contrast, the rooted ring shown in figure 2*C* requires just four gene gain sites, one for each gene, and no gene loss sites. Hence, it is most parsimonious and also far more likely than any tree as will be shown subsequently. In the rooted ring, GGGPS originates from a gene duplication from the ancestral HisF gene that is marked by the labeled pink box at the lower right. From this gene gain site, GGGPS bifurcates and the pink branch on the right flows into the *Halobacteria* (H) and the *Eocyta* (E), while the other half of the pink branch flows toward the top of the ring until it reaches the *Firmicutes* (the *Bacilli*, B, and the *Clostridia*, C). In contrast, the ParC gene gain site originates from a gene duplication from the ancestral GyrA gene. This gene flow, shown in blue on the left side of figure 2*C*, bifurcates and one path flows into the *Actinobacteria* (A) and the DM prokaryotes (D) while the other flows into the *Firmicutes* (B and C). This rooted ring most parsimoniously explains the phylogenetic distributions of the GGGPS and ParC genes using four gene gain sites, including the two gene gain sites at the root for HisF and GyrA, and no gene loss sites. Thus, it explains the distributions of both gene flows better than any tree and also constrains the root to a single site at the bottom of the central black ring. In the next section, we calculate the probability that the rooted ring arose by chance, using hypergeometric distributions, and thereby obtain a statistical estimate of the reliability of this rooted ring.

## Results

### Estimating the Probability of the Root of the Central Ring

To sample the phylogenetic diversity of the GGGPS gene, sequences were downloaded from the Sanger Pfam site. These genes are labeled either GGGPS (at the NCBI site) or PcrB (at the Sanger site). The Sanger site contains 420 aligned sequences and these were used for our analyses. The Sanger Pfam site lists PcrB genes from 111 unique species of *Euryarchaeota* and eocytes, 169 unique species from *Bacilli* and *Clostridia*, and 21 unique species from *Actinobacteria* and DM prokaryotes (0, 2, 12, and 7 species from *Actinobacteria*, *Proteobacteria*, *Flavobacteria*, and *Cytophagia*, respectively).

**Fig. 3.—:**
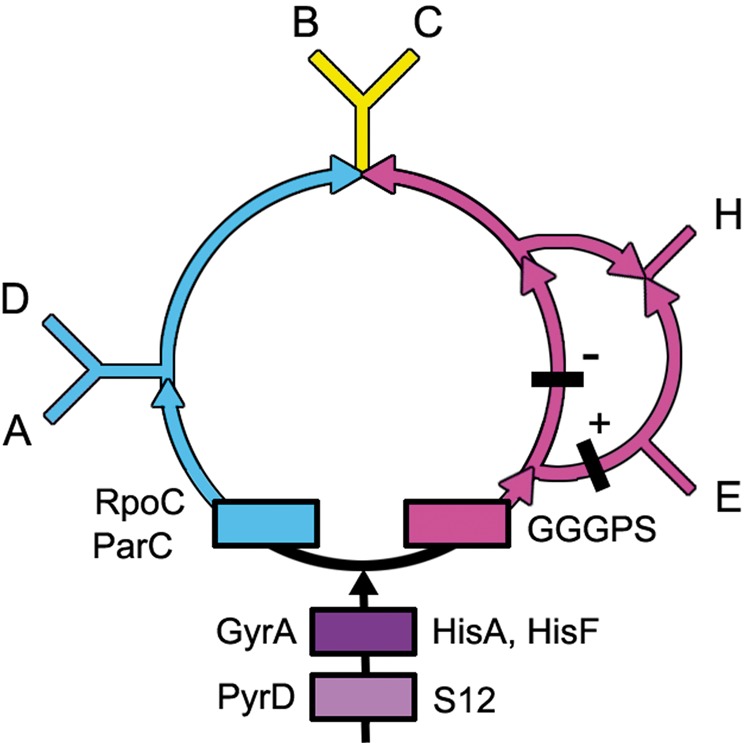
Gene gain sites are shown for eight of the genes analyzed here. Four orthologous genes, PyrD, HisA, HisF, and GGGPS, code for enzymes responsible for building nucleotides, amino acids, and lipids. PyrD codes for the enzyme that performs the final, critical step needed to make the first pyrimidine, Orotate. The gene products of HisA and HisF perform two decisive steps in the synthesis of the amino acid histidine, and GGGPS codes for the penultimate enzyme in the ether lipid biosynthesis pathway. Ribosomal genes S12 and RpoC are orthologous. Ribosomal protein S12 participates in maintaining the fidelity of mRNA translation and the DNA-dependent RNA polymerase, RpoC, transcribes RNAs from DNAs. ParC and GyrA are orthologous and code for topoisomerases. Thus, these genes are representative of the fundamental cellular processes of membrane, protein, RNA, and DNA synthesis. Shown in black are the locations of two GGGPS indels. All the indels within the genes used to construct the rooted central ring support this topology. See supplementary section S2, Supplementary Material online, for gene alignments, orthologs, and indels.

We estimate the statistical support for a GGGPS gene gain site by considering a hypergeometric distribution model (sampling without replacement), that is, a “Two Urn” model. Under this model, we ask whether it is possible that the GGGPS gene flow is larger on the right side of figure 3 than on the left side due to sampling errors. The population size for this test is 420 sequences, the number of successes for the Euryarchaeota and the eocytes in the sample is 111, and the number of successes for the Actinobacteria and the DM prokaryotes is 21. Equal sample sizes, 210, are assumed for both groups. Accordingly, there is no statistical support on the left side of the graph in figure 2*A* for a GGGPS gene gain site, because the cumulative probability for the two-sided test is *P*_Cum_ < 3.9502 × 10^−^^22^. Hence, the GGGPS gene site on the right side of the graph in figure 2*C* is strongly supported.

Statistical support for the ParC gene gain site, on the left side of the graph, is even stronger than that for the GGGPS site. The Sanger Pfam site lists 8,014 sequences for this gene, that is, for ParC, DNA_TopoisoIV, or PF00521. There are 1,505 unique species representing the DM prokaryotes, the *Proteobacteria*, the *Spirochetes*, and the *Cyanobacteria* alone, and ParC genes from the *Firmicutes* are present in 744 unique species. This brings the total for the AD clade to 2,249 unique species. Another 376 uncharacterized prokaryotes and 256 probable DM prokaryotes, i.e., those not included in the table defining the DM taxa in supplementary section S1, Supplementary Material online, are conservatively excluded from this calculation. In comparison, only 39 genes are present from unique euryarchaeal species (*Halobacteria*, *Methanogens*, and relatives). Hence, there is even stronger statistical support (*P*_Cum_ < 10^−^^191^) for the ParC gene gain site being on the left side of the rooted ring than there is for placing the GGGPS gene gain site on the right side of the rooted ring. Accordingly, statistical support for the root of the rings is quite impressive.

The rooted rings that optimally explain the distribution of both the genes and indels, shown in figure 3, are slightly more complex as they accommodate an additional indel in the GGGPS gene that is present in the *Eocyta* and is absent in the *Halobacteria*. This is represented by black bars marked by a minus or a plus. There are no gene loss sites. All indels present within these genes and those discussed in supplementary section S3, Supplementary Material online, support this rooted graph.

### Testing the Topology of the Inner Rooted Rings

**Fig. 4.—:**
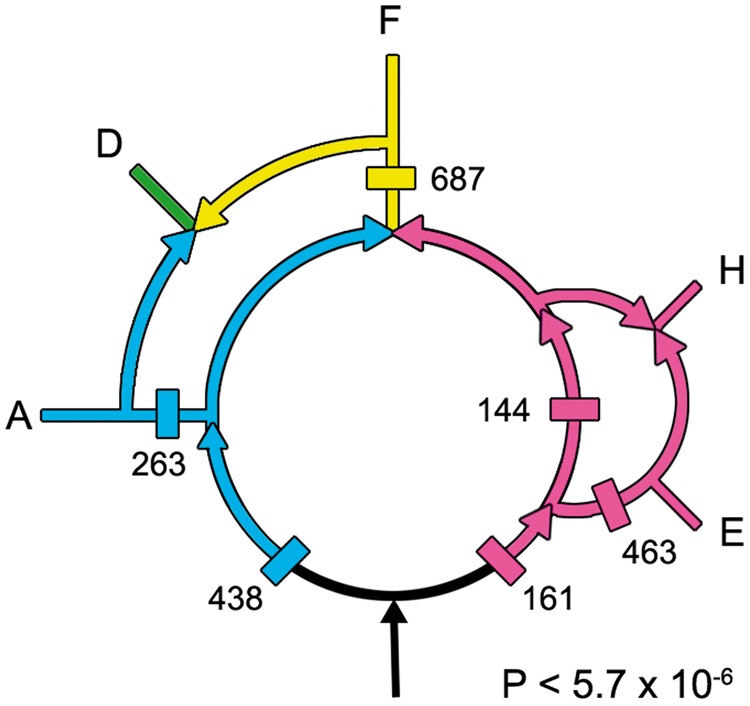
The numbers of genes present in the various gene flows calculated from complete genomes are marked here. See the text for additional details.

It is possible to estimate the statistical support for this inner rooted ring by calculating how many genes have passed through the gene gain sites predicted by the indels. These sites are marked by rectangles in figure 4 and are labeled with the numbers of genes that originated from these sites.

These predicted gene flows were experimentally determined by analyzing complete genomes, rather than indels, and thereby provide an independent estimate of gene flows based on all of the genes that are present within 15 representative whole genomes. In contrast, the rooted ring was reconstructed based on relatively few indels, which nevertheless have the advantage that they are based on sequences present in thousands of species. Although conditioned reconstructions need to be improved (Spencer et al. 2007; McCann et al. 2008), conditioning was not used since the ring derived from indels had strong statistical support for a unique root. Thus, gene presence/absence analyses could be used to test whether both methods support the same rooted rings. Used together, they can provide independent, statistical estimates of the topology of the rooted rings. Whole-genome analyses also provide lists of the genes that are present within each gene flow, and thus can be used to map the origins of cellular processes. In contrast, indels sample diverse populations far better and can root rings and provide high statistical support for individual gene flows. Together, these two methods strongly support the rooted rings, *P* < 7.1 × 10^−^^6^, as described below.

Whole-genome analyses were performed using the OrthoMCL website to test the topology of the rooted rings. Table 3 lists the numbers of genes in each of the phylogenetically informative patterns (+ corresponds to gene present and empty spaces to gene absent). For example, the top entry of table 3 lists the number of genes (263) that are present in the *Actinobacteria*, present in the DM prokaryotes, and absent in the three remaining taxa. The taxa are the *Actinobacteria* (A), DM prokaryotes (D), *Firmicutes* (F), *Halobacteria* (H), and eocytes (E). The six patterns that correspond to the flows that are present in the rings in figure 4 are listed in black, those patterns that are absent from the rings are shown in lavender, and the six largest gene flows in the OrthoMCL analyses are in bold. As one can see, the six largest MCL counts are black and bold, indicating that the indel and OrthoMCL gene flows both support the rooted ring shown in figure 4.

**Table 3:** Numbers of Genes Present in OrthoMCL Analyses

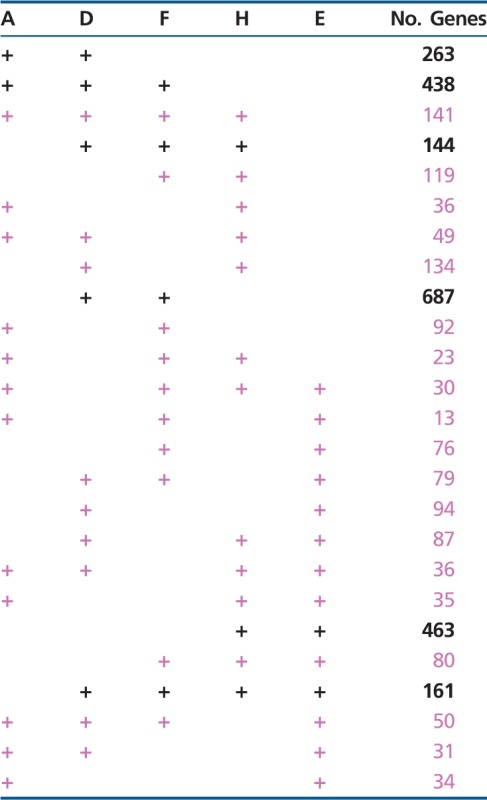

The probability, *P*, that the six predicted gain sites present in figure 4 would by chance correspond to the six largest gene flows in table 3 is calculated to be *P* = (6! 19!)/(25!) <5.7 × 10^−6^. This correspondence between the indel-based and the gene flow-based methods provides robust independent support for the rooted rings presented here.

## Discussion

Whole-genome-based methods can also tell us which genes are being transported through these flows and thereby provide critical information regarding the evolution of fundamental phenotypic and genotypic changes. Thus, they can predict when and how new cellular capabilities evolved.

**Fig. 5.—:**
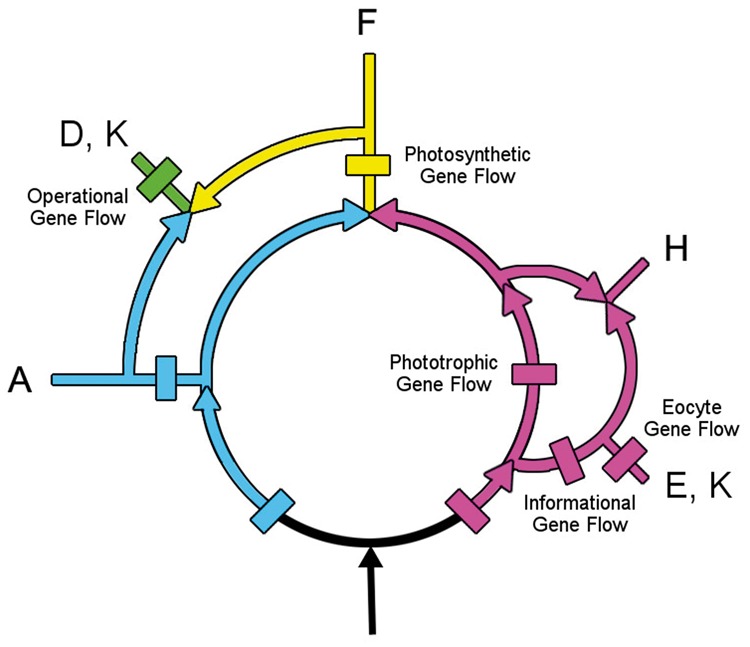
The gene gain sites of the Operational, Informational, Eocyte, Phototropic, and Photosynthetic gene flows present within the rings are mapped here on the rooted ring. Selected genes that help identify function originating within the Phototrophic and the Photosynthetic gene flows are provided in supplementary section S3, Supplementary Material online.

Specifically, these introductions of new gene flows can inform us about major evolutionary innovations. From the gene contents in these flows, we identify five that have been previously noted. These are the Informational (Jain et al. 1999), Operational (Jain et al. 1999), Phototrophic (Nelson-Sathi et al. 2012), Photosynthetic (Lake et al. 1985), and Eocyte (Archibald 2008; Cox et al. 2008; Williams et al. 2012) pathways. Previously, it was not known how these separately proposed pathways were related to each other. In figure 5, they are explicitly mapped onto the rooted rings.

The Operational and Informational gene flows represent two separate paths for genes to flow into eukaryotes. Operational genes are those involved in cellular housekeeping, whereas Informational genes participate in transcription, translation, and related processes (Jain et al. 1999). Informational genes can be readily identified by the large numbers of ribosomal- and RNA-related genes that are present within this flow shown in figure 5. Operational genes, shown in green, are those present in the eukaryotes, which were produced following the fusion/extended symbiosis event(s) that introduced genes from the DM prokaryotes into the eukaryotes.

The Eocyte pathway branches from within the Infomational flow. Like the informational flow, it contains many genes participating in informational processes, but these genes are present only in the two sister taxa, the eukaryotes and the eocytes.

A path for the transfer of more than a thousand genes (Nelson-Sathi et al. 2012) into the *Halobacteria* and the *Eubacteria* has recently been characterized. In their figure 1*B*, this flow is rooted upstream of the *Halobacteria* and the *Eubacteria* and is consistent with the rooting proposed here (see fig. 5). It also parallels the Photocyte pathway that was inferred from phylogenetic analyses of ribosomal structures (Lake 1985). Here, I refer to it as the Phototrophic pathway, because from its start to *Halobacteria*, to the *Firmicutes*, to the DM prokaryotes, and ultimately to the Eukaryotes, it mirrors steps in the evolution of photosynthesis.

Based on the genes present at the beginning of this flow (see supplementary section S3, Supplementary Material online), it introduced many ABC transporters, the C-terminal end of Cytochrome b6, and numerous membrane components that are annotated as being present in the “inner membrane” of the DM prokaryotes. It also introduced spore proteins, like SpoVR, that are responsible for constructing the outer peptidoglycan layer that covers the endospores found within the *Firmicutes*. The Phototrophic pathway laid the foundations for electron transfers and thereby for the subsequent emergence of photophosphorylation via the purple membrane system in the *Halobacteria* (Lake 1985) and subsequently for the origin of photosynthesis in the Firmicute *Heliobacteria*.

The Photosynthetic pathway branches from within the Phototrophic flow. Photosystems I and II developed in the Photosynthetic pathway, as did variations on modes of photosynthesis, including oxygenic photosynthesis. Many novel genes for electron transport were introduced and an abundance of flagellar genes emphasize the importance of motility for efficient photosynthesis.

Because the rings are rooted, they predict that the chronological order of appearance of phototrophic mechanisms (from earliest to most recent) is Photophosphorylation → Photosynthesis-Photosystem I → Oxygenic photosynthesis-Photosystem II. The *Cyanobacteria* are thought to have been present by ∼2.3 Gyr (corresponding approximately to the rise of oxygen in the atmosphere [Bekker 2004]), and thus the Photophosphorylation and Photosynthesis-Photosystem I gene flows arose earlier than ∼2.3 Gyr.

Although the rooted rings allow us to date the relative order of emergence of processes within a single gene flow, like the Phototrophic flow, they do not allow us to date the appearance times of modern phyla which share a common flow. For example, one cannot deduce the relative appearance times of the *Firmicutes* and the *Halobacteria*. This is because even though both phyla originated from within the Photrophic flow, two unrelated gene flows determined when they emerged as phyla. Thus, it is quite possible that the genome merger that produced the *Firmicutes* may have occurred before the genome merger that produced the *Halobacteria* or vice versa.

### The Rings as a Source of Information Regarding the Formation of Phyla

In the rooted rings, the merger of two gene flows can be produced by extended symbioses and by endosymbioses (Lake 2009). Thus, rings have the ability to reveal formative steps in the evolution of life that cannot be obtained from trees. One way to test hypotheses like these is to search for cellular structures that might indicate whether the merger of two large gene flows could have resulted from an extended symbiosis or from an endosymbiosis.

The best-known endosymbionts are the chloroplasts and the mitochondria (Margulis 1970; Dayhoff and Schwartz 1980). Both are surrounded by double membranes. Some eukaryotes have hosted even more disparate organelles, including eukaryotes within eukaryotes, complete with their own organelles and nuclei (Tanifuji et al. 2011). Traditionally, endosymbioses are suspected when multiple gene flows are present, but the presence of additional membranes can confirm them. Highly visible examples include prokaryotic *Buchnera* endosymbionts within some aphids (Moran et al. 2009), the previously mentioned DM prokaryotes, and possibly even the eukaryotic nucleus (Lake 1988; Lake and Rivera 1994). It is also possible that endosymbioses may have occurred in the rooted rings. For example, two separate gene flows merge to form the *Firmicutes.* But unlike the *Halobacteria, the Firmicutes* contain multiple membranes. Specifically, the *Firmicutes* possess unique DM-bounded candidate “organelles.”

These are the endospores (Errington 2003) which are produced in response to starvation in the *Bacilli* and the *Clostridia*. During sporulation, the mother cell differentiates into two morphologically distinct parts and produces an asymmetrically positioned septum adjacent to one pole of the cell that appears to be similar to, but is different from, that found in normal divisions (Higgins and Dworkin 2012). In a series of well-documented steps, surprisingly called “engulfment," the membrane of the mother cell surrounds the nascent spore and engulfs it. Subsequently, the processes of cortex synthesis and coat formation complete the development of the spore within the mother cell.

**Fig. 6.—:**
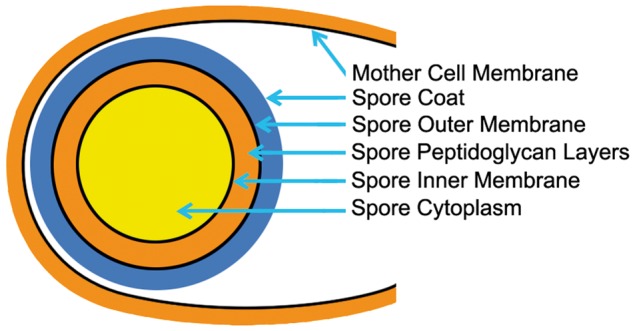
The cellular organization of a sporulating *Bacillus* cell. The spore is shown at the left within its mother cell. The dehydrated cytoplasm of the spore is shaded in yellow. It is surrounded by the spore inner membrane, by complex peptidoglycan layers shown in orange, by the spore outer membrane, and by a thick proteinaceous spore coat shown in blue. The spore resides within the mother cell cytoplasm. The mother cell will subsequently rupture and release the spore which will remain dormant until conditions are right for it to germinate.

As illustrated in figure 6, the spore cortex contains a dehydrated cytoplasm, surrounded by a cytoplasmic membrane, which is surrounded by differentiated peptidoglycan layers, and by an outer membrane. This is enclosed in a protein spore coat. This DM-bound pre-spore is itself surrounded by the cytoplasm, the outer membrane, and the peptidoglycan layer of the mother cell. Subsequently, the endospore is released from the mother cell into the environment, where it will remain until conditions are right for germination. Together, the DM endospore, the transfer to the *Firmicutes* of SpoR genes through the Phototropic gene flow, and the presence of two independent gene flows into the *Firmicutes* make a good case for an endosymbiotic origin of the *Firmicutes*.

### Evolutionary Biology as Practiced Under the Rooted Rings and Under the Three-Domain Hypothesis

It is helpful to envision what our individual scientific lives might be like when the rooted rings are used as a model for the evolution of life on Earth and to compare this with the way evolutionary and microbial science is performed under the Three-Domains Hypothesis (Woese et al. 1990) and under the Rings Hypothesis (Kuhn 1964).

Science under the 3D hypothesis necessarily consists of placing newly identified organisms into one of the three categories that are used to define all life. Under this system, whenever a new organism is discovered, the first question to ask is whether it is a eukaryote, a bacterium, or an archaeon. There are no alternatives to this evolutionary hegemony, everything needs to fit, or if it does not fit it is inevitably shoehorned into one of the three categories. But everything does not always fit (Archibald 2008; Williams et al. 2012), and that is a problem. As a result, the 3D hypothesis rarely, if ever, provides clues to the evolutionary intermediates that exist between organisms. In fact, it cannot tell us about how one domain evolved from another, because if it did then the two domains would be connected and no longer independent domains. For a similar reason, the 3D model cannot be rooted, because if it were then three domains would become two, since two of the domains would be related by a common root.

Now, imagine how scientific life would be under the Rings Hypothesis. First of all, one would not have to be afraid of discovering connections between taxonomic groups. In fact, it is the gene flows between groups that inform us about the innovations and processes that made them possible. Thus, the discovery of a significant new gene flow does not invalidate the rings but only improves their usefulness. Changes in the rings, when well supported, are natural, positive, and nonthreatening precisely because they can potentially add to our understanding of the evolution of life on Earth. This gives the rooted rings the ability to follow gene flows and pinpoint the beginnings of major events in the evolution of life.

## Supplementary Material

Supplementary sections S1–S4 are available at *Genome Biology and Evolution* online (http://www.gbe.oxfordjournals.org/).

Supplementary Data
