# Supplementary material for: The Deep Roots of the Rings of Life
Source: Genome Biol Evol. 2013 Nov 26;5(12):2440–8. doi: 10.1093/gbe/evt194 (PMC3879980; doi:10.1093/gbe/evt194)
Supplement: Supplementary Data [file supp_5_12_2440__index.html]

The Deep Roots of the Rings of Life — The Deep Roots of the Rings of Life — Supplementary Data 

# The Deep Roots of the Rings of Life

## Supplementary Data

files

**Files in this Data Supplement:**

- Supplementary Data - doc file
