# Supplementary material for: The Deep Roots of the Rings of Life
Source: Genome Biol Evol. 2013 Nov 26;5(12):2440–8. doi: 10.1093/gbe/evt194 (PMC3879980; doi:10.1093/gbe/evt194)
Supplement: Supplementary Data [file supp_evt194_Lake_SupMatFinal.doc]

**Supplementary Information.**

**Section S1. Taxon Selection.**

As described in the text, this study focuses on seven natural, phylogenetically well separated groups: euryarchaeota, “clostridia”, “bacilli”, actinobacteria, double membrane prokaryotes, eocytes, and eukaryotes. Together they encompass all known life. The euryarchaeota are primarily extremophiles surrounded by single membranes; the extended eocytes are the sister group to the eukaryotes, the *Clostridia* are unique among the single membrane prokaryotes for containing photosynthetic organisms as well as fermenting ones; the *Bacilli* include both bacilli and carbohydrate fermenting lactic acid bacteria; the *Actinobacteria*, formerly called the high GC prokaryotes, are morphologically and biochemically diverse and contain many human pathogens; and the double membrane prokaryotes are a speciose, probably primitively photosynthetic, taxon exclusively containing prokaryotes surrounded by double membranes. These 37 known prokaryotic taxa are either listed in Table S1, or discussed below. Furthermore, those groups not included in Table S1, are nevertheless included the statistical analyses within the body of the paper.

Several groups are not listed in Table S1, either because they have not been definitively classified, or because the compositions of the groups have been affected by some recent findings. These groups are the: *Chlorobi, Cytophagia, Flavobacteria, Fibrobacteres, Bacteriodetes, Dictyoglomi, Mollicutes, Thermotoga,* and *Deinococcus*. The *Rubrobacteridae*, formerly in the *Actinobacteria*, are included within the “Clostridia”, consistent with recent phylogenetic analyses[^3-5^](#_ENREF_3). The *Acidimicrobidae* and the *Coriobacteridae*, are tentatively moved to the “Clostridia" based on their positions in principal component analyses[^4^](#_ENREF_4), but the classification of the “Mollicutes” remains in doubt. For eight taxa within the double membrane prokaryotes, there is still relatively little information available. These are: the *Verrucomicrobia*, the *Thermomicrobia*, the *Thermodesulfobacteria*, the *Nitrospirae*, the *Fibrobacteres*, the *Dictyoglomi*, the *Deferribacteres* and the *Chrysiogenetes*.

**Table S1.**

**Actino- Eocyta and Double Membrane Prokaryotes bacteria Clostridia Bacilli Euryarchaeota**

**(Phyla) (Subclasses) (Classes) (Orders) (Classes) .**

Acidobacteria Proteobacteria Actino- Clostridia Bacillales Archaeoglobi

Aquificae Planctomycetes bacteridae (includes Lactobac- Halobacteria

Chloroflexi Spirochaetes Rubrobact- bacillales Methano-

Chlalmydiae Thermodesulfo- eridae, bacteria

Cyanobacteria bacteria Coriobact- Methanopyri Fusobacteria Thermomicrobia eriales Thermococci

Symbio- Thermoplasmata

bacterium Thermoprotei

**Section S2. The Indels supporting the New Rooted Graph of Life.**

The paralogous sets representing the indels present in the eight genes that are informative regarding the new rooted graph of life are summarized in Tables S2A, S2B, and S2C, shown below. Indel **insertions** are shown in **blue**, and **deletions** are shown in red.

**Table S2A. The GyrA/ParC indels.**

**GyrA**

D-Proteobacteria NGSGGIAVGMATNIPPHNLGEVIDGCVALIDNPAIE----LSELMELIPGPDFPT

D-Proteobacteria NGSSGIAVGMATNIPPHNLTEVINGCLAYIDDEDIS----IEGLMEHIPGPDFPT

D-Cyanobacteria NGSSGIAVGMATNIPPHNLGELIDALVAVIHNPEIT----DLELMQYVHGPDFPT

A-Actinobacteria NGSSGIAVGMATNIPTHNLREVNEAVQWSLAHPNAS**HEEL**LEACMERIKGPDFPG

F-Firmicutes NGSTGISSGYATEIPPHNLGEVIDATIYLLKHPNAS----LEDLMNYVKGPDFPT

R-Archaebacteria NGSSGIAVGMSTNIPPHNLGELVDATVHLLGNPDCT----VEDLMEHIKGPDFPT

**ParC**

D-Proteobacteria NGAGGIAVGMATNIPPHNLGEVIDACLLLIDQPDVT----TDQLLDLVPGPDFPT

D-Proteobacteria NGTTGIAVGMATDIPPHNLREVAQAAIALIDQPKTT----LDQLLDIVQGPDYPT

D-Cyanobacteria NGCSGIAVGMATNVPPHNLGEVVDGLIALIDNPDLP----DEKLFQLIPGPDFPT

A-Actinobacteria NGASGIAVGMATNMAPHNLVEVVGAARHLLDNPDAT----LDDLMAYIPGPDLPS

F-Firmicutes NGANGIAVGMTTNIPPHNLSEVISGLHMLMRNPDAT----TKDLMKEIPGPDFPT

R-Euryarchaeota  **---------------** Gene Missing ------------------

E-Eocyta  **---------------** Gene Missing ------------------

**Table S2B. The RpoC/S12 indels.**

**RpoC**

D-Proteobacteria LVKRIEDAGLEKVKIRSVL ------------- TCQSRRG-ICSKCY

D-Proteobacteria LVNKIEQAGIEKLYIRSVL ------------- TCKSRHG-ICATCY

D-Cyanobacteria LSKSFEAAGVKAVSVRSPL ------------- TCEANRS-VCRKCY D-Deino-Thermus LIKAAEAGEVREVPVRSPL ------------- TCQTRYG-VCQKCY

D-Aquificae LAEKITKAGIEKVRVRSPL ------------- TCEAKHG-VCAMCY

A-Actinobacteria LIDALVAHGVEEVKTRSIL ------------- TCESQVG-TCAMCY

F-Firmicutes MARKIVDAGVEEVTIRSVF ------------- TCATRHG-VCRHCY

R-Euryarchaeota -------- Gene Region Missing --------

E-Eocyta -------- Gene Region Missing --------

# S12

D-Proteobacteria LVRKPRVSEKLKSKSPALE **-------------** NCPQRRG**-**VCTRVY

D-Cyanobacteria LIRSERSKVQKKTKSPALK **-------------** QCPQRRG**-**VCTRVY

D-Deino-Thermus LLRKGRKVLQKKSKVPALK **-------------** GSPFRRG**-**VCTVVK

D-Aquificae LVKYGREKRKKKSKAPALQ **-------------** GCPQKRG**-**VCVRVY

A-Actinobacteria LVRKGRQDKVEKNKTPALE **-------------** GSPQRRG**-**VCTRVF

F-Clostridia LVRKGRKTVASKSTAPALK **-------------** ECPQKRG**-**VCTVVK

F-Clost-minor LVRKGRYVEEYKSTAPALQ KGMNTLRKKATDI SAPQKRG**-**VCTSVK

F-Bacilli LIRKGRKAKVKKSDSPALN KGYNSFKKVQTDL SSPQKRG-VCTRVG

R-Halobacteria LKKD**--**RQQRRWSDSEYAR RERGLGKKSDPLE GAPQGRGIVLEKVG

R-Thermoplasmata LLEN**--**RKKFRWSDRDYKR RVLQLKRKSDPLE GAPQAKGIAIEKVG

E-Eocyta LRRK**--**RLKFRWSQREFRI RMLDLKRKYDPLE GAPMARGIVLEKVG

**PyrD Table S2C. The Beta/Alpha8 Indels.**

D-Proteobacteria LQLKSTEIIRRLSLEL**NG**RLPIIGVGGIDSVIA

D-Proteobacteria VREGSTRVIRALCGLL**GD**AVPIIGVGGILAGEH

D-Cyanobacteria LRSRSTEVIRLLHRTT**QG**QLPIIGVGGIFSAED

D-Deino-Thermus LTARSTELVRAAYRLT**RG**RMPIVGVGGIFTAED

D-Other DM Proks ILPIAVRMIYQVYEKF**GD**RIPIIGVGGITTWED

A-Actinobacteria LKARSLEVLSRLYARV**GD**RITLVGVGGIENAED

F-Bacilli IKPVAIRMVHEVSQAV--NIPIIGMGGIETAED

F-Clostridia VKPIALRMVHEVAKTV--DIPVIGLGGISTAED

R-Halobacteria IRERATEQVRFVAERT--DQPVVGVGGVATAED

R-Thermoplasmata IKPVGIRYVYEVKKET--GKEIIGVGGISNYKD

R-Thermoprotei LYPIALRIIKDVYEEY--GVDIIGVGGVYDWTD

HisA

D-Proteobacteria MQGCNVPFTKALAEAT--SIPVIASGGIHNLGD

D-Proteobacteria LQGINIDATVKLAQSV--SIPVIASGGLSSLKD

D-Cyanobacteria LAGPNLAALRSMADAS--TVPVIASGGVGCMAD

D-Deino-Thermus LRGLDRDLMRQVRGLW--HGELIVGGGVADTND

D-Other DM Proks LEGVDVEPYKEIKKHV--KKPVIASGGVSSLED

A-Actinobacteria LGGPNLDLLAGVADRT--DAPVIASGGVSSLDD

F-Bacilli LAGPNVEQLELLQKNV--ATRLIASGGVASIQD

F-Clostridia LKGPNLQAMKEMADSV--SMDVIASGGVSRLKD

R-Halobacteria LDGVRTDPVRRLVDSV--DIPVIASGGVATIND

R-Thermoplasmata NSDGTGHISRIEKFWD--EGYFMYAGGVNSIDD

R-Thermoprotei TKGGIDNNVVEYVKSV--KKIKEYAGGIGSLDD

HisF

D-Proteobacteria KNGFDLGVTRAISDAL--GIPVIASGGVGNLQH

D-Proteobacteria KSGFDLELTRAVSDAV--PVPVIASGGVGNLQH

D-Cyanobacteria QAGYDLELTRAVAQAV--PVPVIASGGAGCLDH

D-Deino-Thermus RAGFDLEATRAVAREV--DLPVIASGGAGKVQD

D-Other DM Proks KDGYDIELNRAISEAV--NIPVIASGGAGKKEH

A-Actinobacteria KAGFDLALLRAVRAAV--TVPVIASGGAGAVEH

F-Bacilli KNGYDLRLTEEISKSV--SVPVIASGGCGHADH

F-Clostridia KDGYDIELTRTVSENV--KIPVIASGGAGKMEH

R-Halobacteria KDGYDIPLMKAVCDTV--STPVIASSGCGSPED

R-Thermoplasmata KKGFDTDLIRKITGSV--NIPVIASGGAGSPED

R-Thermoprotei RLGYDLELTRKIVDSV—-NIPVIASGGAGKMEH

GGGPS

D-Proteobacteria ---------------------------------

D-Proteobacteria ---------------------------------

D-Cyanobacteria ---------------------------------

D-Deino-Thermus ---------- Genes Absent ---------

D-Other DM Proks ---------------------------------

A-Actinobacteria ---------------------------------

B-Bacilli MLGDIEAVKKTKAVLE--TSTLFYGGGIKDAET

C-Clostridia RFGDPAWVGAAAGAMR--GARLFYGGGIGTAEQ

B-Bacilli IYGDVSKVQAVSEHLT--ETQLFYGGGISSEQQ

R-Halobacteria MFGDTEKVQAAHDALD--DATLFYGGGIRDYDA

R-Archaeoglobus IYGNPELVAEVKKVLD--KARLFYGGGIDSREK

R-Methanobacter.. PEHVPEEMIALVKRCT--DQILIVGGGIRSGED

E-Methanocaldoc.. SYPVNNETIALSKKLS--GINIIVGGGIRKPEI

E-Pyrococcus PEPVPEEMVRVVKSVI--DVPLIVGGGIKSGEQ

These alignments and the indels contained within them are fairly well understood, since some have been used in previous, tree-based rootings[^6-11^](#_ENREF_6). But it was not previously known that they contained information that rejected all trees. Only when logical conflicts arose between some indels, did it become apparent that trees could not parsimoniously fit the data. However, it took time and effort to identify additional indels that could distinguish between the alternative possible graphs, such as the GGGPS gene distribution. As a result the statistical significance of GGGPS (Gerenyl-Gerenyl-Glycerol-Phosphate Synthase), a member of the alpha-beta 8 family deserves more discussion, as does that of the ParC gene. These are extensively discussed in the **Results** section of the paper.

**Section S3. Selected Informative Genes within the Phototrophic- and Photosynthetic- Gene Flows.**

Photosynthetic Flow

#60 OG5_129817

# Sequences # Taxa # Match Pairs (%) Ave E-Value Ave % Coverage Ave % Identity

63 60 2664 (136.4%) 4.87e-08 76.6 28.9

Pfam Domain(s): Mitochondrial inner membrane protein;

#79 OG5_130915

# Sequences # Taxa # Match Pairs (%) Ave E-Value Ave % Coverage Ave % Identity

49 39 1411 (119.9%) 8.47e-07 90.8 48.3

Keyword(s): 30S ribosomal protein S21; ribosomal protein; ribosomal protein S21; ribosomal; protein S21; 30S ribosomal;

Pfam Domain(s): Ribosomal protein S21;

#89 OG5_131633

# Sequences # Taxa # Match Pairs (%) Ave E-Value Ave % Coverage Ave % Identity

43 29 1466 (162.3%) 6.75e-08 92.8 28.8

Pfam Domain(s): Bacterial type II secretion system protein F domain;

#90 OG5_131641

# Sequences # Taxa # Match Pairs (%) Ave E-Value Ave % Coverage Ave % Identity

43 24 1716 (190%) 1.00e-07 92.8 34.0

Keyword(s): flagellin;

Pfam Domain(s): Bacterial flagellin C-terminus; Bacterial flagellin N-terminus;

#115 OG5_132474

# Sequences # Taxa # Match Pairs (%) Ave E-Value Ave % Coverage Ave % Identity

37 23 1302 (195.4%) 3.18e-08 93.7 32.7

Pfam Domain(s): Periplasmic binding proteins and sugar binding domain of the LacI family;

#117 OG5_132634

# Sequences # Taxa # Match Pairs (%) Ave E-Value Ave % Coverage Ave % Identity

35 24 1190 (200%) 5.97e-37 93.5 47.2

Keyword(s): ABC transporter; ABC; binding; ATP binding protein; transporter; ATP binding;

Pfam Domain(s): ABC transporter;

#124 OG5_133120

# Sequences # Taxa # Match Pairs (%) Ave E-Value Ave % Coverage Ave % Identity

33 22 1055 (199.8%) 5.79e-28 95.5 48.1

Keyword(s): ABC transporter; ABC; ABC transporter; permease; transporter;

Pfam Domain(s): Binding-protein-dependent transport system inner membrane component;

#134 OG5_133319

# Sequences # Taxa # Match Pairs (%) Ave E-Value Ave % Coverage Ave % Identity

32 31 990 (199.5%) 2.55e-14 91.5 34.3

Keyword(s): cell division protein ftsA; cell division protein; ftsA; cell division protein;

Pfam Domain(s): Cell division protein FtsA;

#139 OG5_133532

# Sequences # Taxa # Match Pairs (%) Ave E-Value Ave % Coverage Ave % Identity

31 22 930 (200%) 1.82e-24 92.9 44.0

Keyword(s): flagellar biosynthesis protein; flagellar biosynthesis protein fliP; protein fliP; flagellar;

Pfam Domain(s): FliP family;

#140 OG5_133533

# Sequences # Taxa # Match Pairs (%) Ave E-Value Ave % Coverage Ave % Identity

31 22 930 (200%) 8.61e-27 96.3 34.5

Keyword(s): flagellar; flagellar biosynthesis protein flhB; protein flhB; flagellar; flhB;

Pfam Domain(s): FlhB HrpN YscU SpaS Family;

#157 OG5_134242

# Sequences # Taxa # Match Pairs (%) Ave E-Value Ave % Coverage Ave % Identity

28 21 756 (200%) 1.93e-43 93.1 48.9

Keyword(s): flagellum specific ATP synthase; flagellum specific;

Pfam Domain(s): ATP synthase alpha/beta family, nucleotide-binding domain;

#177 OG5_135083

# Sequences # Taxa # Match Pairs (%) Ave E-Value Ave % Coverage Ave % Identity

25 23 531 (177%) 9.03e-08 94.7 33.8

Keyword(s): flagellar hook protein; flagellar hook protein; flagellar hook protein flgE; flagellar hook protein flgE;

Pfam Domain(s): Flagella basal body rod protein; Domain of unknown function (DUF1078);

#178 OG5_135087

# Sequences # Taxa # Match Pairs (%) Ave E-Value Ave % Coverage Ave % Identity

25 21 358 (119.3%) 8.45e-08 84.9 32.7

Keyword(s): flagellar motor; flagellar motor protein; motB;

Pfam Domain(s): OmpA family;

#180 OG5_135093

# Sequences # Taxa # Match Pairs (%) Ave E-Value Ave % Coverage Ave % Identity

25 22 505 (168.3%) 2.32e-07 77.9 43.1

Keyword(s): flagellar; flagellar motor switch protein; switch protein; flagellar; switch protein fliN; flagellar motor;

Pfam Domain(s): Surface presentation of antigens (SPOA);

#181 OG5_135094

# Sequences # Taxa # Match Pairs (%) Ave E-Value Ave % Coverage Ave % Identity

25 22 504 (168%) 8.52e-08 83.4 27.1

Keyword(s): flagellar; flagellar; flagellar hook associated protein flgK; flagellar hook associated protein;

Pfam Domain(s): Domain of unknown function (DUF1078);

#189 OG5_135383

# Sequences # Taxa # Match Pairs (%) Ave E-Value Ave % Coverage Ave % Identity

24 23 458 (165.9%) 7.53e-08 66.5 34.4

Keyword(s): transglycosylase; murein transglycosylase; lytic;

Pfam Domain(s): Transglycosylase SLT domain;

#195 OG5_135692

# Sequences # Taxa # Match Pairs (%) Ave E-Value Ave % Coverage Ave % Identity

23 20 350 (138.3%) 1.08e-07 85.5 27.9

Keyword(s): flagellar; flagellar;

Pfam Domain(s): Flagellar hook-associated protein 2 C-terminus;

#198 OG5_135724

# Sequences # Taxa # Match Pairs (%) Ave E-Value Ave % Coverage Ave % Identity

23 23 374 (147.8%) 5.63e-07 66.9 38.1

Keyword(s): septum; inhibitor; septum formation inhibitor;

Pfam Domain(s): Septum formation inhibitor MinC, N-terminal domain; Septum formation inhibitor MinC, C-terminal domain;

#199 OG5_135726

# Sequences # Taxa # Match Pairs (%) Ave E-Value Ave % Coverage Ave % Identity

23 21 463 (183%) 1.18e-07 93.0 34.6

Keyword(s): flagellar motor protein motA; protein motA; flagellar motor protein; motA; motor; flagellar motor;

Pfam Domain(s): MotA/TolQ/ExbB proton channel family;

#200 OG5_135730

# Sequences # Taxa # Match Pairs (%) Ave E-Value Ave % Coverage Ave % Identity

23 22 304 (120.1%) 5.26e-07 87.7 37.1

Keyword(s): flagellar; rod protein; flagellar; flgB; flagellar basal body rod protein flgB; rod protein flgB; rod;

Pfam Domain(s): Flagella basal body rod protein;

#211 OG5_136232

# Sequences # Taxa # Match Pairs (%) Ave E-Value Ave % Coverage Ave % Identity

22 21 387 (167.5%) 1.84e-07 76.5 35.2

Keyword(s): flagellar basal body rod modification protein; flagellar; rod modification protein;

Pfam Domain(s): Flagellar hook capping protein;

#222 OG5_136479

# Sequences # Taxa # Match Pairs (%) Ave E-Value Ave % Coverage Ave % Identity

21 21 420 (200%) 4.49e-15 97.5 37.2

Keyword(s): flagellar; flagellar motor switch protein G; flagellar motor switch protein; switch protein; flagellar motor;

Pfam Domain(s): FliG C-terminal domain;

#223 OG5_136481

# Sequences # Taxa # Match Pairs (%) Ave E-Value Ave % Coverage Ave % Identity

21 20 420 (200%) 2.38e-15 99.0 50.9

Keyword(s): flagellar; rod protein; protein flgG; flagellar; flagellar basal body rod protein; rod protein flgG; basal body; rod;

Pfam Domain(s): Domain of unknown function (DUF1078); Flagella basal body rod protein;

#224 OG5_136484

# Sequences # Taxa # Match Pairs (%) Ave E-Value Ave % Coverage Ave % Identity

21 20 388 (184.7%) 3.84e-08 89.9 31.2

Keyword(s): flagellar ms-ring protein; flagellar; flagellar; ms-ring;

Pfam Domain(s): Secretory protein of YscJ/FliF family; Flagellar M-ring protein C-terminal;

#225 OG5_136485

# Sequences # Taxa # Match Pairs (%) Ave E-Value Ave % Coverage Ave % Identity

21 20 420 (200%) 2.06e-10 96.9 41.8

Keyword(s): flagellar basal body rod protein flgC; flagellar; flagellar; rod protein flgC; flgC; rod;

Pfam Domain(s): Domain of unknown function (DUF1078); Flagella basal body rod protein;

#226 OG5_136487

# Sequences # Taxa # Match Pairs (%) Ave E-Value Ave % Coverage Ave % Identity

21 21 311 (148%) 8.22e-08 91.9 35.9

Keyword(s): flagellar motor switch protein fliM; flagellar motor switch protein; flagellar motor;

Pfam Domain(s): Surface presentation of antigens (SPOA); Flagellar motor switch protein FliM;

#227 OG5_136495

# Sequences # Taxa # Match Pairs (%) Ave E-Value Ave % Coverage Ave % Identity

21 20 403 (191.9%) 3.47e-09 90.2 30.8

Keyword(s): flagellar; flagellar; flagellar biosynthesis protein fliR; fliR; protein fliR;

Pfam Domain(s): Bacterial export proteins, family 1;

#247 OG5_137366

# Sequences # Taxa # Match Pairs (%) Ave E-Value Ave % Coverage Ave % Identity

19 18 285 (166.6%) 9.00e-08 75.3 27.9

Keyword(s): flagellar; flagellar; flagellar hook associated protein flgL; flagellar hook associated protein;

Pfam Domain(s): Bacterial flagellin N-terminus;

#248 OG5_137368

# Sequences # Taxa # Match Pairs (%) Ave E-Value Ave % Coverage Ave % Identity

19 18 282 (164.9%) 3.10e-07 88.4 37.5

Keyword(s): flagellar protein; flagellar protein fliS; flagellar protein; fliS;

Pfam Domain(s): Flagellar protein FliS;

#259 OG5_137870

# Sequences # Taxa # Match Pairs (%) Ave E-Value Ave % Coverage Ave % Identity

18 17 306 (200%) 3.93e-42 99.4 46.5

Keyword(s): oligopeptide; permease; transporter; oligopeptide;

Pfam Domain(s): Binding-protein-dependent transport system inner membrane component;

#261 OG5_137885

# Sequences # Taxa # Match Pairs (%) Ave E-Value Ave % Coverage Ave % Identity

18 18 190 (124.1%) 5.81e-07 88.2 40.5

Keyword(s): flagellar hook-basal body protein; flagellar; protein fliE; flagellar hook-basal body protein fliE; flagellar hook-basal body;

Pfam Domain(s): Flagellar hook-basal body complex protein FliE;

#271 OG5_138303

# Sequences # Taxa # Match Pairs (%) Ave E-Value Ave % Coverage Ave % Identity

17 16 272 (200%) 1.48e-48 96.6 38.1

Keyword(s): ABC transporter; ABC; binding; transporter;

Pfam Domain(s): ABC transporter transmembrane region;

#272 OG5_138346

# Sequences # Taxa # Match Pairs (%) Ave E-Value Ave % Coverage Ave % Identity

17 14 272 (200%) 2.72e-24 98.1 44.9

Keyword(s): antiporter; arginine/ornithine antiporter;

Pfam Domain(s): Amino acid permease;

#274 OG5_138465

# Sequences # Taxa # Match Pairs (%) Ave E-Value Ave % Coverage Ave % Identity

17 16 245 (180.1%) 4.25e-08 90.6 40.6

Keyword(s): periplasmic precursor protein; bifunctional; 2,3-cyclic nucleotide 2-phosphodiesterase/3-nucleotidase;

Pfam Domain(s): 5'-nucleotidase, C-terminal domain;

#276 OG5_138552

# Sequences # Taxa # Match Pairs (%) Ave E-Value Ave % Coverage Ave % Identity

17 14 272 (200%) 7.35e-31 88.5 50.0

Keyword(s): cytochrome; oxidase subunit ii; subunit ii; ii; ubiquinol oxidase; oxidase;

Pfam Domain(s): Cytochrome C oxidase subunit II, periplasmic domain; COX Aromatic Rich Motif;

#277 OG5_138562

# Sequences # Taxa # Match Pairs (%) Ave E-Value Ave % Coverage Ave % Identity

17 15 272 (200%) 1.63e-51 92.6 50.1

Keyword(s): ABC transporter; glycine; glycine; transporter;

Pfam Domain(s): Binding-protein-dependent transport system inner membrane component;

#282 OG5_138780

# Sequences # Taxa # Match Pairs (%) Ave E-Value Ave % Coverage Ave % Identity

17 15 255 (187.5%) 2.56e-07 93.6 46.5

Keyword(s): iv; cytochrome; ubiquinol oxidase; oxidase; ubiquinol oxidase subunit; subunit iv;

Pfam Domain(s): Prokaryotic Cytochrome C oxidase subunit IV;

#294 OG5_139295

# Sequences # Taxa # Match Pairs (%) Ave E-Value Ave % Coverage Ave % Identity

16 14 222 (185%) 6.57e-09 89.4 29.3

Keyword(s): ABC transporter; transporter;

Pfam Domain(s): Binding-protein-dependent transport system inner membrane component;

#305 OG5_140175

# Sequences # Taxa # Match Pairs (%) Ave E-Value Ave % Coverage Ave % Identity

15 14 180 (171.4%) 1.51e-07 91.7 37.0

Keyword(s): hypothetical protein;

Pfam Domain(s): ABC transporter substrate binding protein;

#306 OG5_140185

# Sequences # Taxa # Match Pairs (%) Ave E-Value Ave % Coverage Ave % Identity

15 15 111 (105.7%) 1.09e-07 95.6 38.7

Keyword(s): flagellar; protein fliL; fliL; flagellar basal body associated protein fliL;

Pfam Domain(s): Flagellar basal body-associated protein FliL;

#322 OG5_141271

# Sequences # Taxa # Match Pairs (%) Ave E-Value Ave % Coverage Ave % Identity

14 14 134 (147.2%) 4.16e-07 84.9 41.2

Pfam Domain(s): Cytochrome C assembly protein;

#330 OG5_141700

# Sequences # Taxa # Match Pairs (%) Ave E-Value Ave % Coverage Ave % Identity

14 14 182 (200%) 7.13e-28 88.9 54.2

Keyword(s): ABC transporter; permease; transporter;

Pfam Domain(s): Binding-protein-dependent transport system inner membrane component;

#349 OG5_143161

# Sequences # Taxa # Match Pairs (%) Ave E-Value Ave % Coverage Ave % Identity

13 12 140 (179.4%) 6.43e-08 88.0 37.6

Keyword(s): ABC transporter; binding protein; binding; transporter;

Pfam Domain(s): Bacterial extracellular solute-binding proteins, family 5 Middle;

#369 OG5_144466

# Sequences # Taxa # Match Pairs (%) Ave E-Value Ave % Coverage Ave % Identity

12 11 132 (200%) 1.78e-25 93.7 46.4

Keyword(s): ABC transporter; ABC; ATP binding protein;

Pfam Domain(s): ABC transporter;

#370 OG5_144467

# Sequences # Taxa # Match Pairs (%) Ave E-Value Ave % Coverage Ave % Identity

12 12 132 (200%) 1.37e-07 94.1 43.4

Keyword(s): ABC transporter; ATP binding protein;

Pfam Domain(s): ABC transporter;

#371 OG5_144468

# Sequences # Taxa # Match Pairs (%) Ave E-Value Ave % Coverage Ave % Identity

12 11 128 (193.9%) 8.97e-08 85.0 36.0

Keyword(s): binding; transporter;

Pfam Domain(s): Periplasmic binding protein;

#372 OG5_144471

# Sequences # Taxa # Match Pairs (%) Ave E-Value Ave % Coverage Ave % Identity

12 9 132 (200%) 9.74e-33 99.1 50.8

Keyword(s): nitrate reductase; gamma subunit; respiratory nitrate reductase;

Pfam Domain(s): Nitrate reductase gamma subunit;

#401 OG5_146671

# Sequences # Taxa # Match Pairs (%) Ave E-Value Ave % Coverage Ave % Identity

11 11 104 (189%) 1.77e-08 87.8 33.9

Keyword(s): binding protein; ABC transporter; iron;

#465 OG5_153454

# Sequences # Taxa # Match Pairs (%) Ave E-Value Ave % Coverage Ave % Identity

9 7 72 (200%) 6.62e-16 89.3 38.2

Keyword(s): ABC transporter; amino acid;

Pfam Domain(s): Binding-protein-dependent transport system inner membrane component;

#482 OG5_153711

# Sequences # Taxa # Match Pairs (%) Ave E-Value Ave % Coverage Ave % Identity

9 9 60 (166.6%) 3.02e-09 93.2 47.3

Keyword(s): str; electron transport complex; electron transport complex protein rnfG;

Pfam Domain(s): FMN-binding domain;

#483 OG5_153784

# Sequences # Taxa # Match Pairs (%) Ave E-Value Ave % Coverage Ave % Identity

9 9 72 (200%) 3.86e-47 97.7 51.0

Keyword(s): str; electron transport complex; electron transport complex protein rnfD;

Pfam Domain(s): NQR2, RnfD, RnfE family;

#490 OG5_155530

# Sequences # Taxa # Match Pairs (%) Ave E-Value Ave % Coverage Ave % Identity

8 8 56 (200%) 1.25e-22 93.9 43.3

Keyword(s): ABC transporter; ABC transporter; permease;

Pfam Domain(s): Binding-protein-dependent transport system inner membrane component;

#525 OG5_159035

# Sequences # Taxa # Match Pairs (%) Ave E-Value Ave % Coverage Ave % Identity

7 7 40 (190.4%) 5.01e-09 92.0 36.0

Keyword(s): str; channel;

Pfam Domain(s): Voltage gated chloride channel;

#550 OG5_160814

# Sequences # Taxa # Match Pairs (%) Ave E-Value Ave % Coverage Ave % Identity

7 7 42 (200%) 7.14e-16 92.7 48.3

Keyword(s): str; inner membrane protein;

Pfam Domain(s): YeeE/YedE family (DUF395);

#577 OG5_164873

# Sequences # Taxa # Match Pairs (%) Ave E-Value Ave % Coverage Ave % Identity

6 5 28 (186.6%) 8.21e-11 92.0 31.1

Keyword(s): ABC transporter; binding protein; iron;

Pfam Domain(s): Periplasmic binding protein;

#582 OG5_164896

# Sequences # Taxa # Match Pairs (%) Ave E-Value Ave % Coverage Ave % Identity

6 6 30 (200%) 1.70e-08 89.9 67.1

Keyword(s): str;

Pfam Domain(s): SNARE associated Golgi protein;

#590 OG5_164960

# Sequences # Taxa # Match Pairs (%) Ave E-Value Ave % Coverage Ave % Identity

6 6 30 (200%) 1.35e-15 81.7 31.1

Keyword(s): sporulation protein; stage ii sporulation; sporulation; stage ii sporulation protein D;

Pfam Domain(s): Stage II sporulation protein;

#615 OG5_171034

# Sequences # Taxa # Match Pairs (%) Ave E-Value Ave % Coverage Ave % Identity

5 5 20 (200%) 3.04e-36 94.8 46.0

Keyword(s): transporter; nickel transporter; transporter permease;

Pfam Domain(s): Binding-protein-dependent transport system inner membrane component;

#668 OG5_191280

# Sequences # Taxa # Match Pairs (%) Ave E-Value Ave % Coverage Ave % Identity

3 3 6 (200%) 1.00e-21 90.3 27.7

Keyword(s): str; ABC transporter; membrane;

Pfam Domain(s): Binding-protein-dependent transport system inner membrane component;

Phototrophic Flow

#23 OG5_128441

Keyword(s): ABC transporter; ATP binding protein; transporter; ATP binding; ATP;

Pfam Domain(s): ABC transporter;

#30 OG5_129259

Keyword(s): ABC transporter; ABC; amino; permease; transporter;

Pfam Domain(s): Binding-protein-dependent transport system inner membrane component;

#31 OG5_129334

# Sequences # Taxa # Match Pairs (%) Ave E-Value Ave % Coverage Ave % Identity

71 29 4970 (200%) 4.40e-12 96.2 36.9

Keyword(s): ABC transporter; ABC; ATP binding protein; transporter; ATP binding;

Pfam Domain(s): ABC transporter;

#48 OG5_131894

# Sequences # Taxa # Match Pairs (%) Ave E-Value Ave % Coverage Ave % Identity

41 30 1308 (159.5%) 1.21e-07 81.9 33.3

Keyword(s): ABC transporter; binding protein; ABC; binding; transporter; periplasmic;

Pfam Domain(s): Bacterial extracellular solute-binding protein;

#50 OG5_132141

# Sequences # Taxa # Match Pairs (%) Ave E-Value Ave % Coverage Ave % Identity

39 25 1481 (199.8%) 4.09e-13 92.4 37.0

Keyword(s): ABC transporter; ABC; transporter;

Pfam Domain(s): Binding-protein-dependent transport system inner membrane component;

#55 OG5_132434

# Sequences # Taxa # Match Pairs (%) Ave E-Value Ave % Coverage Ave % Identity

37 25 1332 (200%) 1.15e-15 84.1 38.0

Keyword(s): ABC transporter; ABC; permease; transporter;

Pfam Domain(s): Binding-protein-dependent transport system inner membrane component;

#64 OG5_133115

# Sequences # Taxa # Match Pairs (%) Ave E-Value Ave % Coverage Ave % Identity

33 31 984 (186.3%) 8.33e-09 96.0 54.9

Keyword(s): rod shape-determining protein; mreB; rod shape-determining protein; rod; rod;

Pfam Domain(s): MreB/Mbl protein;

#76 OG5_134302

# Sequences # Taxa # Match Pairs (%) Ave E-Value Ave % Coverage Ave % Identity

28 20 516 (136.5%) 1.36e-07 79.8 29.7

Keyword(s): ABC transporter; binding protein; binding; transporter; periplasmic;

Pfam Domain(s): Bacterial extracellular solute-binding protein;

#81 OG5_134712

# Sequences # Taxa # Match Pairs (%) Ave E-Value Ave % Coverage Ave % Identity

26 17 648 (199.3%) 2.39e-08 79.5 29.6

Keyword(s): ABC transporter;

Pfam Domain(s): ABC transporter;

#88 OG5_135741

# Sequences # Taxa # Match Pairs (%) Ave E-Value Ave % Coverage Ave % Identity

23 19 458 (181%) 5.78e-08 89.3 31.6

Keyword(s): ABC transporter; transporter;

Pfam Domain(s): Branched-chain amino acid transport system / permease component;

#92 OG5_136087

# Sequences # Taxa # Match Pairs (%) Ave E-Value Ave % Coverage Ave % Identity

22 19 434 (187.8%) 4.42e-08 93.2 35.9

Keyword(s): ABC transporter; permease; permease protein;

Pfam Domain(s): Branched-chain amino acid transport system / permease component;

#95 OG5_136660

# Sequences # Taxa # Match Pairs (%) Ave E-Value Ave % Coverage Ave % Identity

21 17 302 (143.8%) 3.05e-07 82.6 35.8

Keyword(s): monovalent cation/H+ antiporter subunit G; antiporter; antiporter subunit G; monovalent;

Pfam Domain(s): Na+/H+ antiporter subunit;

#98 OG5_136945

# Sequences # Taxa # Match Pairs (%) Ave E-Value Ave % Coverage Ave % Identity

20 14 377 (198.4%) 8.12e-09 76.8 35.5

Keyword(s): ABC; permease;

Pfam Domain(s): Binding-protein-dependent transport system inner membrane component;

#105 OG5_137711

# Sequences # Taxa # Match Pairs (%) Ave E-Value Ave % Coverage Ave % Identity

18 16 306 (200%) 6.54e-17 93.1 42.5

Keyword(s): ATP;

Pfam Domain(s): ABC transporter;

#112 OG5_138519

# Sequences # Taxa # Match Pairs (%) Ave E-Value Ave % Coverage Ave % Identity

17 15 239 (175.7%) 2.51e-10 93.1 30.9

Keyword(s): ABC transporter; ABC; permease; permease protein;

Pfam Domain(s): Binding-protein-dependent transport system inner membrane component;

#114 OG5_139055

# Sequences # Taxa # Match Pairs (%) Ave E-Value Ave % Coverage Ave % Identity

16 15 228 (190%) 5.02e-08 58.4 33.4

Keyword(s): ATP dependent; helicase hepA; ATP dependent; helicase; ATP dependent helicase;

Pfam Domain(s): Helicase conserved C-terminal domain;

#120 OG5_144456

# Sequences # Taxa # Match Pairs (%) Ave E-Value Ave % Coverage Ave % Identity

12 11 117 (177.2%) 2.90e-08 87.6 31.4

Keyword(s): hypothetical protein;

Pfam Domain(s): SNARE associated Golgi protein;

#125 OG5_145082

# Sequences # Taxa # Match Pairs (%) Ave E-Value Ave % Coverage Ave % Identity

12 12 132 (200%) 9.20e-18 86.3 44.8

Pfam Domain(s): SpoVR like protein;

#128 OG5_146609

# Sequences # Taxa # Match Pairs (%) Ave E-Value Ave % Coverage Ave % Identity

11 10 70 (127.2%) 1.24e-06 81.4 53.4

Pfam Domain(s): Cytochrome b(C-terminal)/b6/petD;

#130 OG5_146677

# Sequences # Taxa # Match Pairs (%) Ave E-Value Ave % Coverage Ave % Identity

11 10 61 (110.9%) 3.02e-08 86.2 29.1

Keyword(s): polysaccharide biosynthesis; str; biosynthesis;

Pfam Domain(s): Polysaccharide biosynthesis protein;

#137 OG5_149543

# Sequences # Taxa # Match Pairs (%) Ave E-Value Ave % Coverage Ave % Identity

10 10 41 (91.1%) 7.50e-07 73.5 30.1

Keyword(s): transferase;

Pfam Domain(s): Glycosyl transferase family 2;

#147 OG5_163250

# Sequences # Taxa # Match Pairs (%) Ave E-Value Ave % Coverage Ave % Identity

6 6 30 (200%) 2.00e-32 92.8 46.0

Keyword(s): ABC transporter; amino acid;

Pfam Domain(s): Branched-chain amino acid transport system / permease component;

#148 OG5_187390

# Sequences # Taxa # Match Pairs (%) Ave E-Value Ave % Coverage Ave % Identity

3 3 6 (200%) 3.19e-78 97.3 39.3

Keyword(s): hypothetical protein; str;

Pfam Domain(s): Short chain fatty acid transporter;

**Section S4. Gene Presence/Absence Analyses.**

Presence/Absence analyses were performed using the OrthoMCL website to test the topology of the Rooted Rings. The following table lists the numbers of genes in each of the directly phylogenetic patterns (+ corresponds to gene present, and a blank to gene absent). The taxa are: Actinobacteria (A), Double Membrane Prokaryotes (D), Firmicutes (F), Halobacteria (H), and Eocytes (E). The OrthoMCL queries are: A: mtup+mlep, D: atum+ecol+syne, F: bant+cbot+saur, H: halo+hwal+aful; and E: ssol+cmaq+hbut+msed. The gene flows predicted from the Indel-based, rooted ring are shown in red. The probability, P, that the six predicted flows would correspond by chance to the six largest counts from this set of 25 presence/absence patterns is P < 5.7 x 10^-6^, as described in the text. Thus the results of the whole genome studies and the gene distribution studies are mutually consistent.

**References.**

1 Williams, T. A., Foster, P. G., Nye, T. M. W., Cox, C. J. & Embley, T. M. A congruent phylogenomic signal places eukaryotes within the Archaea. *Proc. R. Soc. B-Biol. Sci.* **279**, 4870-4879, doi:10.1098/rspb.2012.1795 (2012).

2 Halanych, K. M. *et al.* Evidence from 18s Ribosomal DNA That the Lophophorates Are Protostome Animals Inarticulate. *Science* **267**, 1641-1643 (1995).

3 Ohno, M. *et al.* Symbiobacterium thermophilum gen. nov., sp. nov., a symbiotic thermophile that depends on co-culture with a Bacillus strain for growth. *International Journal of Systematic and Evolutionary Microbiology* **50**, 1829-1832 (2000).

4 Garrity, G. & Holt, J. G. in *Bergey's Manual of Systematic Bacteriology* (eds D. Boone & R. W. Castenholz) (Springer, 2001).

5 Wu, M. *et al.* Life in Hot Carbon Monoxide: The Complete Genome Sequence of *Carboxydothermus hydrogenoformans* Z-2901. *PLoS Genetics* **1**, 563-574 (2005).

6 Lake, J. A., Servin, J. A., Herbold, C. W. & Skophammer, R. G. Evidence Excluding the Root of the Tree of Life from the Actinobacteria and the Double Membrane Prokaryotes. *Syst. Biol.* (2008).

7 Lake, J. A., Servin, J. A., Herbold, C. W. & Skophammer, R. G. Evidence for a new root of the tree of life. *Systematic Biology* **57**, 835-343 (2008).

8 Lake, J. A., Skophammer, R. G., Herbold, C. W. & Servin, J. A. Genome beginnings: Rooting the tree of life. *Proc. R. Soc. Lond. B.* **364**, 2177-2185 (2009).

9 Servin, J. A., Herbold, C. W., Skophammer, R. G. & Lake, J. A. Evidence excluding the root of the tree of life from the Actinobacteria. *Mol. Biol. Evol.* **24**, 1-4 (2007).

10 Skophammer, R. G., Herbold, C. W., Rivera, M., Servin, J. A. & Lake, J. A. Evidence that the root of the tree of life is not within the Archaea. *Mol. Biol. Evol.* **23**, 1-4 (2006).

11 Skophammer, R. G., Servin, J. A., Herbold, C. W. & Lake, J. A. Evidence for a Gram positive, Eubacterial Root of the Tree of Life. *Mol. Biol. Evol.* **24**, 1-8 (2007).
